# Characterization of Polyethylene-*Graft*-Sulfonated Polyarylsulfone Proton Exchange Membranes for Direct Methanol Fuel Cell Applications

**DOI:** 10.3390/membranes5040875

**Published:** 2015-12-04

**Authors:** Hyung Kyu Kim, Gang Zhang, Changwoo Nam, T.C. Mike Chung

**Affiliations:** Department of Materials Science and Engineering, The Pennsylvania State University, University Park, PA 16802, USA; E-Mails: tcc3@psu.edu (H.K.K.); guz6@psu.edu (G.Z.); cun120@psu.edu (C.N.)

**Keywords:** polyethylene, direct methanol fuel cells, surface hydrophobicity, electrochemical stability, methanol permeability

## Abstract

This paper examines polymer film morphology and several important properties of polyethylene-*graft*-sulfonated polyarylene ether sulfone (PE-g-s-PAES) proton exchange membranes (PEMs) for direct methanol fuel cell applications. Due to the extreme surface energy differences between a semi-crystalline and hydrophobic PE backbone and several amorphous and hydrophilic s-PAES side chains, the PE-g-s-PAES membrane self-assembles into a unique morphology, with many proton conductive s-PAES channels embedded in the stable and tough PE matrix and a thin hydrophobic PE layer spontaneously formed on the membrane surfaces. In the bulk, these membranes show good mechanical properties (tensile strength >30 MPa, Young’s modulus >1400 MPa) and low water swelling (λ < 15) even with high IEC >3 mmol/g in the s-PAES domains. On the surface, the thin hydrophobic and semi-crystalline PE layer shows some unusual barrier (protective) properties. In addition to exhibiting higher through-plane conductivity (up to 160 mS/cm) than in-plane conductivity, the PE surface layer minimizes methanol cross-over from anode to cathode with reduced fuel loss, and stops the HO• and HO_2_• radicals, originally formed at the anode, entering into PEM matrix. Evidently, the thin PE surface layer provides a highly desirable protecting layer for PEMs to reduce fuel loss and increase chemical stability. Overall, the newly developed PE-g-s-PAES membranes offer a desirable set of PEM properties, including conductivity, selectivity, mechanical strength, stability, and cost-effectiveness for direct methanol fuel cell applications.

## 1. Introduction

Direct methanol fuel cells (DMFCs), in which liquid methanol is used as the fuel, present many advantages, including the ease of transportation and storage, stable fuel under application environments, high energy density, is inexpensive and readily available [1,2]. They are particularly targeted to replace rechargeable batteries for a broad range of portable electronic devices [3,4], such as cell phones, two-way radios, laptop computers, military equipment, *etc.* However, there are several challenges in the large-scale commercialization of DMFCs [4,5]. First, methanol crossover from the anode to cathode occurs during the operation of DMFCs. This phenomenon causes the loss of fuel before it produces protons at the anode and oxidation of methanol in the cathode, leading to the lower efficiency of the whole system [6,7]. Therefore, DMFCs usually require thicker membranes, compared to hydrogen proton exchange membrane fuel cells (PEMFCs). Second, kinetics in the anode are relatively slow, compared to proton exchange membrane fuel cells (PEMFCs), which means low power density, thus increasing the need of a precious catalyst such as platinum [8]. Finally, chemical stability is not well achieved with currently-developed membranes for DMFCs including Nafion^®^ and aromatic hydrocarbon polymers [9,10,11].

Nafion^®^ is the benchmark of PEMs with high proton conductivity and good chemical stability [12,13]. However, in addition to high cost, Nafion^®^ suffers from a high methanol crossover; methanol loss is more than 40% and an abrupt decrease in conductivity caused by the degradation of the sulfonic acid group (CF_2_-SO_3_H) at the end of the side chain under low humidity and high temperature conditions [9,14]. Some research efforts have been made to prepare Nafion composite membranes with various inorganic materials [15,16]. On the other hand, many alternative polymers, including poly(arylene ether sulfone), poly(etherketone), polyimides [17], polybenzimidazole (PBI) [18], and polymer blends [19,20,21,22], have been studied for direct methanol fuel cells with some limited success. In these aromatic polymers, the hydroxyl (HO•) and hydroperoxyl radical (HOO•) originally formed at the anode can attack double bonds in aromatic rings, causing a ring opening or chain scission [9,10,11]. Scientifically, it is very difficult to have a new PEM material with the required high hydrophilicity for proton conductivity while limiting the diffusion of methanol and both (OH•) and (HOO•) radicals. There is a clear need to redesign an appropriate PEM membrane that could simultaneously exhibit high proton conductivity, low methanol crossover, good chemical stability, long term durability, and low cost.

**Scheme 1:**
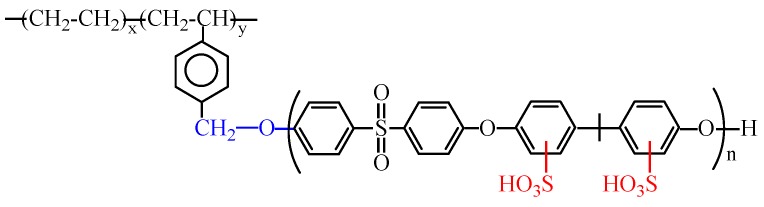
Molecular structure of PE-g-s-PAES graft copolymer.

In our previous paper [23], we discussed the synthesis of a polyethylene-*graft*-sulfonated poly(arylene ether sulfone) (PE-g-s-PAES) graft copolymer that contains a semi-crystalline and hydrophobic PE backbone and several amorphous and hydrophilic s-PAES side chains, as illustrated in Scheme 1. We examined some of its related PEMFC properties, including water swelling, ion exchange capacity, and proton conductivity. It would be scientifically interesting to extend the study in order to examine the associated properties for direct methanol fuel cells (DMFCs), especially the major issues surrounding methanol fuel crossover and polymer stability under an electrochemical processes [24,25]. It is also interesting to note that PE is the most stable polymer, which is commonly used as the container material for strong acids, strong bases, and peroxides. Under free radical conditions, most polymers degrade and reduce their mechanical strength and PE chains engage with a cross-linking reaction and strengthen their mechanical strength.

## 2. Results and Discussion

For a systematic study, a set of PE-g-s-PAES graft copolymers, containing a polyethylene (PE) backbone (molecular weight = 390 k) and various numbers of sulfonated poly(arylene ether sulfone) (s-PAES) side chains (molecular weight = 20 k), were synthesized and cased into PEM membranes (thickness 20–40 μm). Their detailed molecular structures are illustrated and summarized in Table 1. In all PE-g-s-PAES graft copolymers, the degree of sulfonation in s-PAES side chains is very high, in the range of 1.6–1.7 sulfonic acid per arylene ether sulfone unit, and the ion exchange capacity (IEC) in the s-PAES phase is in the range of 3.39–3.67 mmol/g. However, the overall IEC value in each PE-g-s-PAES is dependent on the s-PAES content. With the number of s-PAES side chains increasing from 4.6 to 14.5 (run A-4) per PE backbone, the volume and weight fractions of s-PAES in the graft copolymer change from 25% to 51% and 30% to 57%, respectively, and the IEC value changes from 1.10 to 1.93 (run A-4). For side-by-side comparison, a standard Nafion 117 and two sulfonated poly(arylene ether sulfone) with 30% (BPSH30) and 40% (BPSH40) sulfonation levels, with less than 1 sulfonic acid per arylene ether sulfone unit, are also shown in Table 1. Both BPSH30 and BPSH40 are based on the same polymer with similar molecular weight located in the side chains of the PE-g-s-PAES graft copolymers, however their sulfonation levels are less than half of the s-PAES side chains in the graft copolymers. In fact, the s-PAES side chains should be water soluble if they were not bonded to the PE backbone. As shown in Table 1, the PE-g-s-PAES PEMs show not only similar hydration numbers with BPSH30 and 40 BPSH40 but also excellent mechanical strength. The PE semi-crystalline/hydrophobic backbone in the PE-g-s-PAES graft copolymers clearly provides a unique strong and stable matrix that effectively limits water swelling.

### 2.1. Morphology of PE-g-s-PAES PEMs

**Figure 1:**
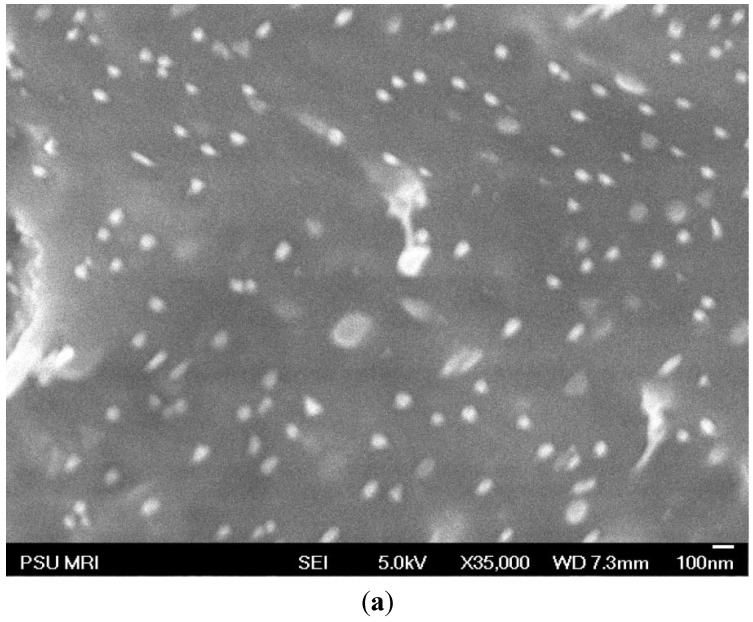

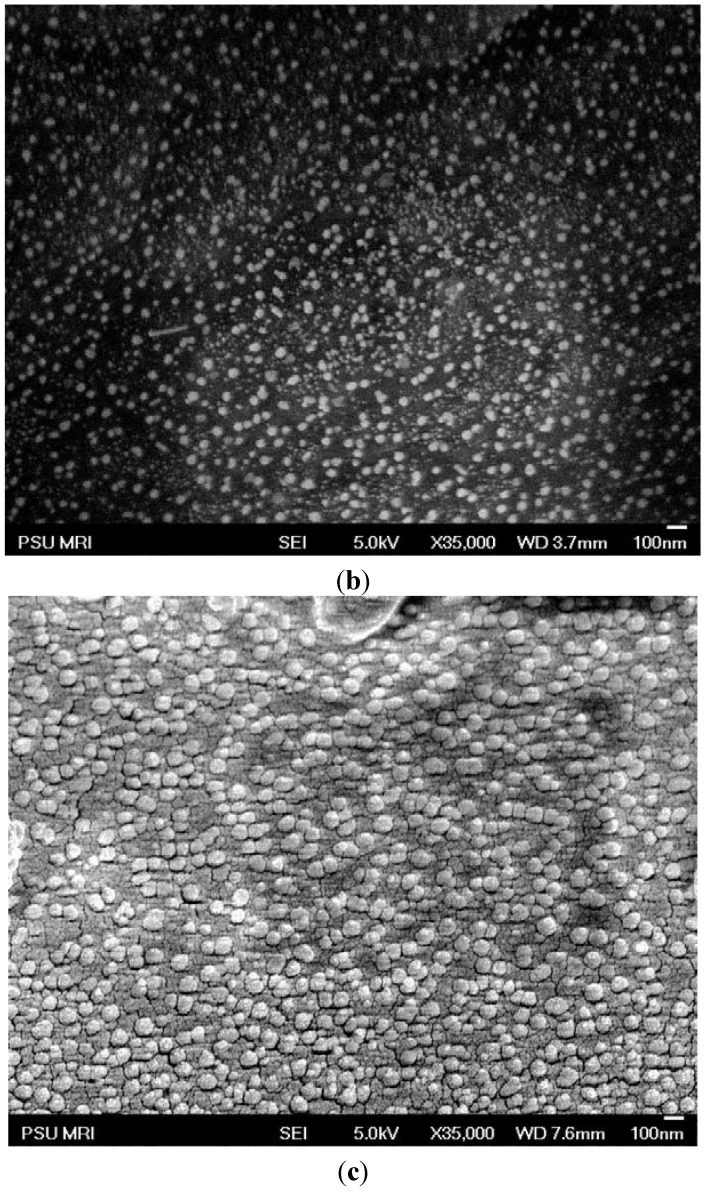
SEM micrographs of two PE-g-s-PAES membranes including (**a**) surface and (**b**) bulk of Sample A-2, and (**c**) the bulk of Sample A-4 in Table 1.

As discussed, with the extreme differences in surface energy between the hydrophobic/crystalline PE backbone and hydrophilic/amorphous s-PAES side chains in the PE-g-s-PAES graft copolymer, it is logical to expect that all solution-casted PE-g-s-PAES membranes shall show a clear self-assembled two-phase morphology with micro-phase separation. In the bulk, the s-PAES side chains shall form a conducting phase in the PE matrix of the PEMs, and the phase changes from discrete domains to continuous channels based on volume fraction. It is very interesting to examine their surface morphology, which shall favor the PE domains with low surface energy (48, 49). Figure 1a,b compares two SEM images of the surface and a cross-section of a PE-g-s-PAES membrane (Sample A-2). Indeed, there are far fewer s-PAES spherical domains (less volume fraction), compared to the dense and uniform s-PAES spherical domains in the bulk. Figure 1c shows the bulk (cross-section) morphology of Sample A-4. Comparing two bulk morphologies between Figure 1b and 1c, it is clear that the increase of the s-PAES content in the graft copolymer increases the size of spherical particles and its overall volume fraction, and the conducting s-PAES domains become a well-connected conducting phase in Sample A-4.

**Table 1:** Illustration and summary of molecular structure, ion exchange capacity, hydration number, mechanical properties of PE-g-s-PAES PEMs, and the comparison with Nafion 117, and two BPSH30 and BPSH40 PEMs.

Sample	No. of s-PAES Per PE	s-PAES (vol %/wt %)	IEC (mmol/g)		Hydration Number (λ)	Tensile Strength (Mpa)	Young’s Modulus (Mpa]	Elongation at Break (%)
			s-PAE	PE-g-s-PAES				
A-1	4.6	25/30	3.67	1.10	11	22 ± 5	870 ± 100	20.8 ± 0.3
A-2	7.7	35/42	3.43	1.44	12	29 ± 5	1150 ± 100	10.8 ± 0.3
A-3	9.4	40/47	3.45	1.62	12	33 ± 5	1290 ± 100	7.2 ± 0.3
A-4	14.5	51/57	3.39	1.93	13	35 ± 5	1480 ± 100	5.7 ± 0.3
BPSH30	–	–	1.3	1.3	10	–	–	–
BPSH40	–	–	1.7	1.7	13	–	–	–
Nafion117	–	–	0.91	0.91	15	14 ± 2	120 ± 10	208 ± 13

**Figure 2:**
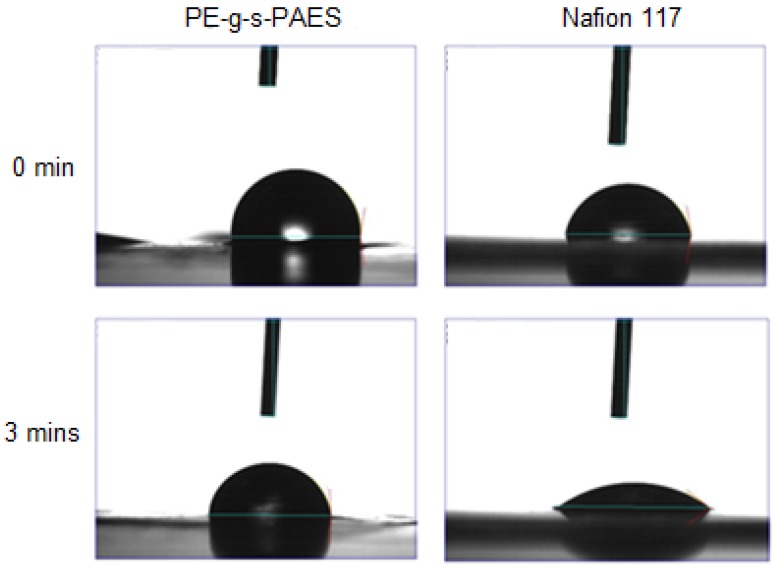
Comparison of water drop on PE-g-s-PAES and Nafion 117 membranes.

Contact angle measurement, using a water drop on PEM surfaces, was also performed to confirm the observed surface morphology. Figure 2 compares the water drop on the membrane surface (*vs.* time) between the PE-g-s-PAES membrane (Sample A-2) and Nafion 117 membrane. Upon the water drop making contact with the Nafion 117 surface, the water drop was immediately diffused into the PEM matrix. After 3 min, the water drop nearly disappeared from the surface, and the water contact angle decreased to 38.03°. On the other hand, the water drop was quite stable on the PE-g-s-PAES PEM surface; it only allows the water drop slowly diffuse into the matrix. Under the same 3 min condition, the contact angle only slightly decreases from 98.41° to 86.61°. Evidently, the PE-g-s-PAES surface is significantly more hydrophobic than the Nafion 117 surface. A thin PE rich layer must cover the majority of the PE-g-PAES membranes’ surface, which results in slow water diffusion through the surface layer.

### 2.2. Methanol Permeability and Selectivity of PE-g-s-PAES PEMs

It is interesting to understand the effects of this unique PEM morphology to several essential properties for direct methanol fuel cells (DMFCs), including in-plane and through-plane proton conductivities, methanol selectivity, and membrane stability under an oxidative operational environment. Table 2 shows the comparison of in-plane and through-plane conductivities, methanol permeability, and selectivity between four PE-g-s-PAES PEMs and three control PEMs, discussed in Table 1. Evidently, the existence of a thin hydrophobic PE-rich layer on the PE-g-s-PAES PEM surface results in the anisotropy, slightly reducing in-plane conductivity, but not through-plane conductivity. The thickness of the PE-rich layer must be very thin (on a molecular length-scale), and the minor component of the s-PAES domains on the surface slowly gives way for the proton to diffuse into the s-PAES hydrophilic channels to maintain high conductivity. Basically, the proton conductivity is proportional to the IEC value. Most of the PE-g-s-PAES membranes achieve high proton conductivity in the same range or higher than Nafion 117.

**Figure 3:**
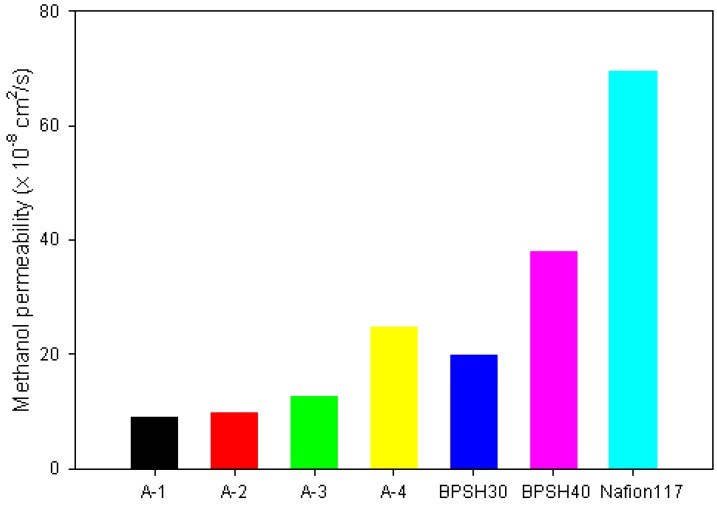
Methanol permeability of PE-g-s-PAES and reference PEMs for DMFCs.

Figure 3 compares methanol permeability for the same set of PE-g-s-PAES PEMS with BPSH 30, BPSH 40, and Nafion 117 reference PEMs (Table 2). All PE-g-s-PAES PEMs show significantly lower methanol permeability than Nafion 117, which proportionally increases with the IEC value of PE-g-s-PAES PEM. Despite a relatively low IEC value (0.91 mmol/g), Nafion 117 shows many times higher methanol permeability, due to its poor phase separated morphology. Furthermore, comparing A-3 PEM with BPSH40, having a similar IEC value, methanol permeability of the A-3 PEM is three times lower than that of BPSH40. These comparative results clearly indicate the benefit of PE-g-s-PAES PEM with its unique morphology. The hydrophobic and semi-crystalline PE-rich surfaces shall substantially prevent the methanol permeation, without much reduction on proton conductivity.

**Table 2:** Comparison of conductivity and permeability of PE-g-s-PAES and reference PEMs.

Sample	In-Plane Conductivity (mS/cm)	Through-Plane Conductivity (mS/cm)	Methanol Permeability (×10^−8^ cm^2^/s)	Selectivity
A-1	30	34	9.02	3.77
A-2	42	57	9.87	5.78
A-3	63	85	12.7	6.69
A-4	85	131	24.9	5.26
BPSH30	30	–	20	1.5
BPSH40	75	–	38	1.97
Nafion 117	77	81	69.7	1.16

Selectivity (defined by the ratio of proton conductivity/methanol permeability) of PEM is an important indicator in determining the applicability in DMFCs. The combination of low methanol permeability and high proton conductivity positions PE-g-s-PAES PEMs with significantly higher selectivity than three reference PEMs (Table 2). In particular, A-3 PEM shows the maximum selectivity of 6.69 that is about six times higher than that of Nafion 117. As discussed previously, these superior properties may be attributed to the combination of surface hydrophobicity and well-organized microphase separated bulk morphology. The surface hydrophobicity shall prevent a rapid absorption of methanol aqueous solution into the PE-g-s-PAES PEMs. The entirely hydrophobic polyethylene matrix may be also responsible for the delay of methanol permeation to some extent since the water repelling region in the membrane will not allow methanol to go through. Many researchers reported that methanol permeability and proton conductivity are proportional to IEC of PEMs [20,21,26,27]. In this case, the PE-g-s-PAES PEMs follows the same general trend. However, it is worthy to note that A-4 PEM abruptly increases methanol permeability, which may be due to the change of the s-PAES hydrophilic phase. Based on our previous report [23], the percolation threshold for the s-PAES domains in the PE-g-s-PAES PEM is at the IEC value ~1.4 mmol/g. In this study, the abrupt increase of methanol permeability appears at around IEC~1.6 mmol/g. The cause of this discrepancy may be associated with the methanol transport in PEM, not by a Grotthus or “hopping” mechanism, but by just by a vehicle mechanism, unlike the proton transport mechanism [28].

### 2.3. Chemical Stability of PE-g-s-PAES PEMs

PEM durability has been recognized as one of the most important issues in PEMFC applications. The Fenton test [29,30,31] has been commonly used to study the long term chemical stability of PEMs, which involves hydrogen peroxide and the ferrous ion (Fenton reagent) redox reaction to produce hydroxyl radicals in an aqueous solution. Similar radical species are formed in the anode [32] of fuel cells, which show aggressive oxidative degradation to many PEM polymers at fast rates [9,14]. In this study, the polymer films with a thickness of 20–40 μm were immersed in an aqueous solution containing 3 wt % H_2_O_2_ and 4 ppm Fe^+2^ ion concentration at 80 and 95 *°*C, respectively, for 1 h. Table 3 summarizes the weight loss of several polymer membranes, including PE-g-s-PAES PEMs, several precursor polymers formed during the preparation of the PE-g-s-PAES polymer, and some common and reported PEM polymers.

Based on the weight loss results, all PE copolymers show excellent oxidative stability under Fenton test conditions. Although the increase of sulfonic acid groups, PE-g-s-PAES PEMs show a slow increase of weight loss. Overall, all PE-g-s-PAES PEMs exhibit superior stability compared to other PEMs [33,34,35], including Nafion 117 that is well appreciated for its chemical stability. Considering the fact that PE-based bottles and containers are commonly used for the storage of strong acids and bases, PE polymer chains shall exhibit excellent stability (inertness) to most aggressive chemicals. Under the free radical condition at high temperatures, the PE polymer chains are not degraded; in fact they form a crosslinking network to produce strong PE materials. Again, the combination of the hydrophobic polyethylene (PE) surface and the matrix in PE-g-s-PAES PEMs must provide effective protection to the hydrophilic s-PAES domains by physically surrounding them; thus, sulfonic acid groups are not exposed to the hydroxyl radicals as much as random copolymers, including Nafion 117.

**Table 3:** Summary of chemical stability of PE-g-s-PAES and reference PEMs.

Sample	IEC (mmol/g)	Residual Weight % after Fenton Test for 1 h	
		80 °C	95 °C
PE-co-p-MS	–	100	100
PE-co-p-MS-Br	–	100	100
PE-g-PAES	–	100	100
PE-g-s-PAES, A-1 (30 wt %)	1.1	100	99.6
PE-g-s-PAES, A-3 (47 wt %)	1.62	98.7	96.9
PE-g-s-PAES, A-4 (56 wt %)	1.93	97.5	95.2
Nafion 117	0.91	96.4	93.7
X30Y8	1.62	40	–

## 3. Experimental Section

### 3.1. Materials and Instrumentation

The PE-g-s-PAES graft copolymers and the corresponding PEM membranes were prepared following the same procedures described in our previous paper [23]. After the sulfonation reaction, the PE-g-s-PAES membranes were kept in deionized (DI) water for characterizations. Three reference polymers, including Nafion 117, and two sulfonated poly(arylene ether sulfone) with 30% (BPSH30) and 40% (BPSH40) sulfonation levels, were used as received. Methanol with a high purity grade (99.9%), 30 weight % hydrogen peroxide aqueous solution, and Iron (II) persulfate heptahydrate (ACS reagent) were used as received. In-plane conductivity was measured by electrochemical impedance spectroscopy (EIS) with a Solartron 1260 frequency response analyzer (Leicester, UK) and through-plane conductivity was measured by Gamry electrochemical measurement system (Philadelphia, PA, USA) in the frequency range of 10 to 1 MHz. The contact angle measurement was performed using Perkin Elmer (Waltham, MA, USA) contact angle goniometry. Surface morphology was observed by scanning electron microscopy (SEM) using Philips XL30 equipment (Beaverton, OR, USA).

### 3.2. Membrane Characterization

Water uptake (%) is calculated by [(W_wet_-W_dry_)/W_dry_] × 100%; wherein W_wet_ is the weight of fully hydrated membrane, and W_dry_ is the weight of completely dried membrane. The membrane was dried under vacuum for 12 h at 70 °C and stored in a desiccator before measuring the weight in “dry” state (W_dry_). The membrane was then equilibrated in de-ionized water for 24 h at room temperature. After blotting the surface of the film quickly, the weight in “wet” state (W_wet_) was measured. IEC was determined by back-titration using 0.01 N NaOH aqueous solution. The PE-g-s-PAES membrane was soaked into 1 M NaCl aqueous solution to release protons from the membrane. The solution was replaced every 4 h. After 24 h, the collected NaCl (aq) solution was titrated with 0.01 N NaOH (aq) using phenolphthalein as an indicator. Colorless NaCl (aq) solution was changed to purple when a certain amount (V_NaOH_) of NaOH (aq) was added. As a reference, the pure 1 M NaCl aqueous solution was titrated using the same condition to obtain the reference amount (V_pure_) of NaOH (aq). IEC (mmol/g) was calculated by [NaOH] × (V_NaOH_-V_pure_)/W_dry_. With the obtained water uptake and IEC value, the hydration number (λ) was calculated by [water uptake (%) × 10]/[IEC value × 18].

### 3.3. Methanol Permeability

**Figure 4:**
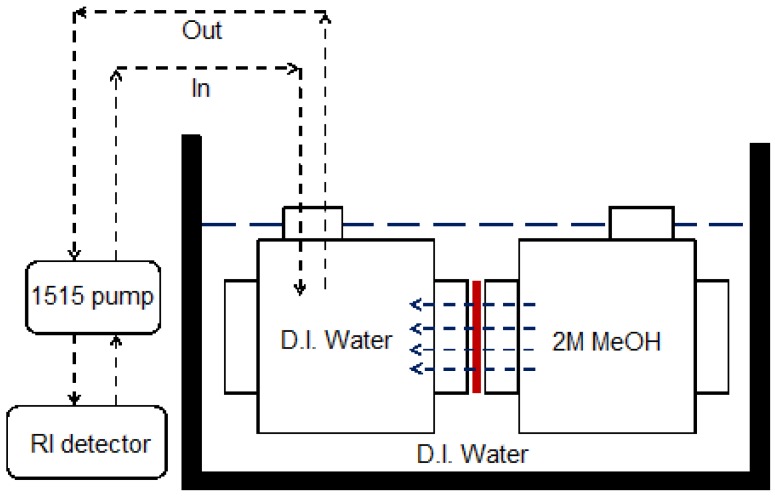
Schematics of two chambers for methanol permeability measurement.

Methanol permeability was measured using a Waters 1515 Pump and Waters 2414 Refractive Index (RI) Detector. The procedure for the measurement is described as follows. The membrane sample is placed between two empty chambers (Figure 4) and clamped to prevent leaking during the measurement. In one chamber, deionized (DI) water is filled and in the other chamber, 2 M methanol aqueous solution is filled. Once the two chambers are filled time is recorded and change of concentration in the chamber in which DI water was filled is monitored by RI detector. The obtained RI values were converted into a concentration of the solution using a regression curve of concentration *vs.* del RI, in which del RI is deference in the refractive index (for more information, see Supporting Information). Methanol permeability was calculated [24] by the equation, $\text{ln}\frac{{\text{M}}_{\text{R}.\text{t}}-{\text{M}}_{\text{L}.\text{t}}}{{\text{M}}_{\text{R}.\text{o}}-{\text{M}}_{\text{L}.\text{o}}}=-\text{DH χ t}$, in which M_R.t_ is the concentration of methanol (MeOH) in methanol solution chamber at time t, M_L.t_ is the concentration of MeOH in the DI water chamber at time t, M_R.o_ is the concentration of MeOH in the methanol solution chamber at t = 0, M_L.o_ is the concentration of MeOH in the DI water chamber at t = 0, D is diffusion coefficient, H is the partition coefficient, DH is permeability, and χ is the geometric parameter (=A/δ [1/V_R_ + 1/V_L_], wherein A is surface area, δ is thickness of membrane, and V_R_ and V_L_ are the volume of the methanol solution chamber and DI water chamber, respectively). The methanol permeability of the Nafion 117 membrane was measured before all measurements in order to validate the experiment condition. The measurement was done within 3 h for each run.

### 3.4. Electrochemical Stability

Electrochemical stability of the PE-g-s-PAES membrane was evaluated by the Fenton test. Iron (II) persulfate heptahydrate (FeSO_4_•7H_2_O) was dissolved in 3% hydrogen peroxide (H_2_O_2_) aqueous solution and the pre-weighed PE-g-s-PAES membrane was immersed in the solution at 80 or 95 °C. The experiment was conducted for an hour, followed by removing the film from the Fenton reagent. The PE-g-s-PAES membrane was thoroughly washed with deionized water and dried in a vacuum at 60 °C for 48 h. The dried membrane was weighed and the electrochemical stability was calculated with the equation, (W_B_ − W_A_)/W_B_ × 100 (%), wherein W_B_ and W_A_ are the weight of the PE-g-s-PAES membrane before and after the Fenton test, respectively. 

## 4. Conclusions

We have systematically studied a set of PE-g-s-PAES PEMs with the objective of determining several essential PEM properties (*i.e.*, conductivity, selectivity, and stability) for direct methanol fuel cells and to understand their structure-property relationship. Compared to Nafion 117 and several common PEM polymers, PE-g-s-PAES PEMs consistently show higher selectivity and higher oxidative stability under elevated temperatures. All experimental results, with a combination of anisotropic proton conductivity, low surface energy by water contact angle, and different SEM micrographs on surface and bulk, indicate a PE-rich hydrophobic surface lay (with molecular scale thickness) and the well-defined microphase separated bulk in PE-g-s-PAES PEMs. With the combination of excellent mechanical strength and limited water swelling reported in the previous paper, the newly developed PE-g-s-PAES PEMs showed very promising properties for DMFCs.

## Supplementary Files

Supplementary File 1

## References

[1] James L., Andrew D. (2003). Fuel Cell Systems Explained.

[2] Apanel G., Johnson E. (2004). Direct methanol fuel cells—Ready to go commercial?. Fuel Cells Bull..

[3] (2004). US Army lab awards MTI Micro DMFC contract. Fuel Cells Bull..

[4] Suominen. A., Tuominen A. (2010). Analyzing the direct methanol fuel cell technology in portable applications by a historical and bibliometric analysis. J. Bus. Chem..

[5] Kamarudin S.K., Achmad F., Daud W.R.W. (2009). Overview on the application of direct methanol fuel cell (DMFC) for portable electronic devices. Int. J. Hydrogen Energy.

[6] Ren X., Springer T.E., Zawodzinski T.A., Gottesfeld S. (2000). Methanol transport through nation membranes. electro-osmotic drag effects on potential step measurements. J. Electrochem. Soc..

[7] Heinzel A., Barragán V.M. (1999). A review of the state-of-the-art of the methanol crossover in direct methanol fuel cells. J. Power Sources.

[8] Shrivastava N.K., Thombre S.B. (2011). Barriers to commercialization of passive direct methanol fuel cells: A review. Int. J. Eng. Sci. Technol..

[9] Curtin D.E., Lousenberg R.D., Henry T.J., Tangeman P.C., Tisack M.E. (2004). Advanced materials for improved PEMFC performance and life. J. Power Sources.

[10] Hubner G., Roduner E. (1999). EPR investigation of HO/radical initiated degradation reactions of sulfonated aromatics as model compounds for fuel cell proton conducting membranes. J. Mater. Chem..

[11] Kaczmarek H., Lindén L.Å., Rabek J.F. (1995). Photo-oxidative degradation of poly(2,6-dimethyl-1,4-phenylene oxide) in the presence of concentrated hydroxy peroxide: The role of hydroxy (HO.) and hydroperoxy (HO_2_.) radicals. Polymer Degrad. Stab..

[12] Steele B.C.H., Heinzel A. (2001). Materials for fuel-cell technologies. Nature.

[13] Mauritz K.A., Moore R.B. (2004). State of understanding of Nafion. Chem. Rev..

[14] Pianca M., Barchiesi E., Esposto G., Radice S. (1999). End groups in fluoropolymers. J. Fluor. Chem..

[15] Park H.S., Kim Y.J., Hong W.H., Lee H.K. (2006). Physical and electrochemical properties of Nafion/polypyrrole composite membrane for DMFC. J. Membr. Sci..

[16] Wang C., Chalkova E., Lee J.K., Fedkin M.V., Komarneni S., Lvov S.N. (2011). Composite membranes with sulfonic and phosphonic functionalized inorganics for reduced relative humidity PEM fuel cells. J. Electrochem. Soc..

[17] Woo Y., Oh S.Y., Kang Y.S., Jung B. (2003). Synthesis and characterization of sulfonated polyimide membranes for direct methanol fuel cell. J. Membr. Sci..

[18] Wang J.T., Wainright J.S., Savinell R.F., Litt M. (1996). A direct methanol fuel cell using acid-doped polybenzimidazole as polymer electrolyte. J. Appl. Electrochem..

[19] Kim D.H., Choi J., Hong Y.T., Kim S.C. (2007). Phase separation and morphology control of polymer blend membranes of sulfonated and nonsulfonated polysulfones for direct methanol fuel cell application. J. Membr. Sci..

[20] Kim H., Kim D., Choi J., Kim S. (2011). Compositional effect on the properties of sulfonated and nonsulfonated polymer blend membranes for direct methanol fuel cell. Macromol. Res..

[21] Kwon Y.H., Kim S.C., Lee S.-Y. (2009). Nanoscale phase separation of sulfonated poly(arylene ether sulfone)/poly(ether sulfone) semi-IPNs for DMFC membrane applications. Macromolecules.

[22] Choi J., Kim D.H., Kim H.K., Shin C., Kim S.C. (2008). Polymer blend membranes of sulfonated poly(arylene ether ketone) for direct methanol fuel cell. J. Membr. Sci..

[23] Kim H.K., Zhang M., Yuan X., Lvov S.N., Chung T.C.M. (2012). Synthesis of polyethylene-based proton exchange membranes containing PE backbone and sulfonated poly(arylene ether sulfone) side chains for fuel cell applications. Macromolecules.

[24] Hickner M.A., Fujimoto C.H., Cornelius C.J. (2006). Transport in sulfonated poly(phenylene)s: Proton conductivity, permeability, and the state of water. Polymer.

[25] Kim Y.S., Wang F., Hickner M., McCartney S., Hong Y.T., Harrison W., Zawodzinski T.A., McGrath J.E. (2003). Effect of acidification treatment and morphological stability of sulfonated poly(arylene ether sulfone) copolymer proton-exchange membranes for fuel-cell use above 100 °C. J. Polymer Sci. Part B.

[26] Kim Y.S., Hickner M.A., Dong L., Pivovar B.S., McGrath J.E. (2004). Sulfonated poly(arylene ether sulfone) copolymer proton exchange membranes: Composition and morphology effects on the methanol permeability. J. Membr. Sci..

[27] Kim J., Kim B., Jung B. (2002). Proton conductivities and methanol permeabilities of membranes made from partially sulfonated polystyrene-block-poly(ethylene-ran-butylene)-block-polystyrene copolymers. J. Membr. Sci..

[28] Pivovar B.S., Wang Y., Cussler E.L. (1999). Pervaporation membranes in direct methanol fuel cells. J. Membr. Sci..

[29] Maletzky P., Bauer R., Lahnsteiner J., Pouresmael B. (1999). Immobilisation of iron ions on nafion^®^ and its applicability to the photo-fenton method. Chemosphere.

[30] Pozio A., Silva R.F., de Francesco M., Giorgi L. (2003). Nafion degradation in PEFCs from end plate iron contamination. Electrochim. Acta.

[31] Fenton H.J.H. (1894). LXXIII—Oxidation of tartaric acid in presence of iron. J. Chem. Soc. Trans..

[32] Schmidt T.J., Paulus U.A., Gasteiger H.A., Behm R.J. (2001). The oxygen reduction reaction on a Pt/carbon fuel cell catalyst in the presence of chloride anions. J. Electroanal. Chem..

[33] Bae B., Yoda T., Miyatake K., Uchida H., Watanabe M. (2010). Proton-conductive aromatic ionomers containing highly sulfonated blocks for high-temperature-operable fuel cells. Angew. Chem. Int. Ed..

[34] Yin Y., Du Q., Qin Y., Zhou Y., Okamoto K.-I. (2011). Sulfonated polyimides with flexible aliphatic side chains for polymer electrolyte fuel cells. J. Membr. Sci..

[35] Chikashige Y., Chikyu Y., Miyatake K., Watanabe M. (2005). Poly(arylene ether) ionomers containing sulfofluorenyl groups for fuel cell applications. Macromolecules.

