# Supplementary material for: Characterization of Polyethylene-Graft-Sulfonated Polyarylsulfone Proton Exchange Membranes for Direct Methanol Fuel Cell Applications
Source: Membranes (Basel). 2015 Dec 4;5(4):875–87. doi: 10.3390/membranes5040875 (PMC4704017; doi:10.3390/membranes5040875)
Supplement: Supplementary File 1 [file membranes-05-00875-s001.pdf]

Supplemental Information

Determination of Methanol Concentration from Refractive Index.

A calibration curve of methanol concentration vs. RI value was pre-determined by several reference solutions. The experimental results are shown in the following Table S1 and Figure S1.

Table S1. Summary of RI value for five methanol concentrations in DI water.

Run	Methanol Concentration in Deionized Water (M)	del RI Value
1	0	-1.55×10^{-7}
2	0.005	3.14×10^{-6}
3	0.01	6.55×10^{-6}
4	0.02	1.32×10^{-5}
5	0.04	2.61×10^{-5}

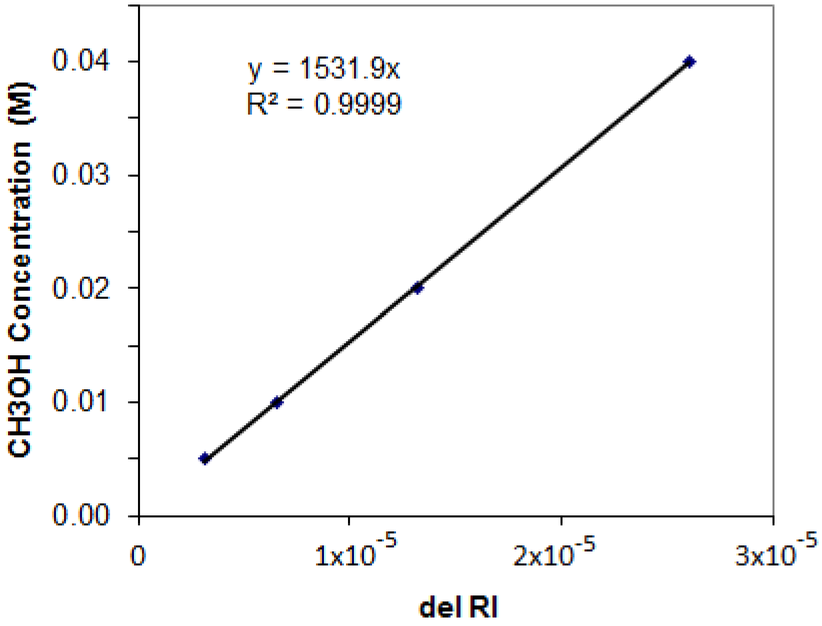

Figure S1. Plot of methanol concentration vs. del RI value.
